# Gene signature predictive of hepatocellular carcinoma patient response to transarterial chemoembolization

**DOI:** 10.7150/ijbs.39534

**Published:** 2019-10-03

**Authors:** Valerie Fako, Sean P. Martin, Yotsawat Pomyen, Anuradha Budhu, Jittiporn Chaisaingmongkol, Sophia Franck, Joyce Man-Fong Lee, Irene Oi-Lin Ng, Tan-To Cheung, Xiyang Wei, Niya Liu, Junfang Ji, Lei Zhao, Zhaogang Liu, Hu-Liang Jia, Zhao-You Tang, Lun-Xiu Qin, Roman Kloeckner, Jens Marquardt, Tim Greten, Xin Wei Wang

**Affiliations:** 1 Laboratory of Human Carcinogenesis;; 2 Liver Cancer Program, Center for Cancer Research, National Cancer Institute, Bethesda, Maryland, USA;; 3 Departments of Pathology;; 4 Surgery, and State Key Laboratory for Liver Research, The University of Hong Kong, Hong Kong, China;; 5 Life Sciences Institute, Zhejiang University, Hangzhou, China;; 6 Shandong Cancer Hospital and Institute, Jinan, China;; 7 Fudan University, Shanghai, China;; 8 University Medical Center Mainz, Mainz, Germany;; 9 Thoracic and GI Malignancies Branch, Center for Cancer Research, National Cancer Institute, Bethesda, Maryland, USA

**Keywords:** Transarterial Chemoembolization, hepatocellular carcinoma, precision oncology, gene signature, treatment response, hypoxia signaling

## Abstract

Transarterial chemoembolization (TACE) is a commonly used treatment modality in hepatocellular carcinoma (HCC). The ability to identify patients who will respond to TACE represents an important clinical need, and tumor gene expression patterns may be associated with TACE response. We investigated whether tumor transcriptome is associated with TACE response in patients with HCC. We analyzed transcriptome data of treatment-naïve tumor tissues from a Chinese cohort of 191 HCC patients, including 105 patients who underwent TACE following resection with curative intent. We then developed a gene signature, TACE Navigator, which was associated with improved survival in patients that received either adjuvant or post-relapse TACE. To validate our findings, we applied our signature in a blinded manner to three independent cohorts comprising an additional 130 patients with diverse ethnic backgrounds enrolled in three different hospitals who received either adjuvant TACE or palliative TACE.

TACE Navigator stratified patients into Responders and Non-Responders which was associated with improved survival following TACE in our test cohort (Responders: 67 months vs Non-Responders: 39.5 months, p<0.0001). In addition, multivariable Cox model demonstrates that TACE Navigator was independently associated with survival (HR: 9.31, 95% CI: 3.46-25.0, p<0.001). In our validation cohorts, the association between TACE Navigator and survival remained robust in both Asian patients who received adjuvant TACE (Hong Kong: 60 months vs 25.6 months p=0.007; Shandong: 61.3 months vs 32.1 months, p=0.027) and European patients who received TACE as primary therapy (Mainz: 60 months vs 41.5 months, p=0.041). These results indicate that a TACE-specific molecular classifier is robust in predicting TACE response. This gene signature can be used to identify patients who will have the greatest survival benefit after TACE treatment and enable personalized treatment modalities for patients with HCC.

## Introduction

Globally, hepatocellular carcinoma (HCC) is the second leading cause of cancer-related mortality, and incidence continues to rise 1-3. Since HCC is clinically and biologically heterogeneous and refractory to most therapy 4, patient outcomes remain poor. For nearly 10 years, sorafenib remained the only approved systemic agent for advanced HCC, providing improvement in overall survival (OS) by only a few months 5. Recently, regorafenib and nivolumab were approved in the second line^6^, ^7^, and lenvatenib as a new first-line treatment 8, although again these drugs offer only modest benefit.

For unresectable patients with localized disease and well-preserved liver function, transarterial chemoembolization (TACE), a percutaneous, image-guided procedure, is the first-line treatment of choice throughout the United States, Europe, and Asia 9-12. Although not utilized in Europe and North America, the use of adjuvant TACE, following resection with curative intent, is common in mainland China, Hong Kong and Taiwan 13-15. However, the ability to establish the efficacy of adjuvant TACE has been met with mixed results in randomized control trials 16, 17. In addition, when TACE is performed in the palliative setting for unresectable patients, only a modest survival improvement is expected. One plausible explanation for the lack of therapeutic efficacy is poor patient selection. Thus, new strategies to stratify patients who will have the greatest survival benefit following TACE is an urgent task for future clinical management of HCC and is a central strategy of precision oncology 18, 19.

We hypothesize that patient outcomes following TACE is related to the underlying transcriptomic profile which can be utilized to predict outcomes in a prospective manor. Because the lack of adequate tumor biospecimens from intermediate HCC patients, due to existing diagnostic guidelines, we first sought to investigate tumor transcriptome from resected patients who also received adjuvant or post-recurrence TACE. To test the robustness of our TACE-associated molecular signature in predicting TACE response in a diagnostic setting, we examined diverse populations of patients with differing HCC etiologies and multiple TACE indications using a Nanostring platform and formalin-fixed paraffin-embedded (FFPE) samples.

## Materials and methods (figure 1)

### Test Cohort

**Figure 1:**
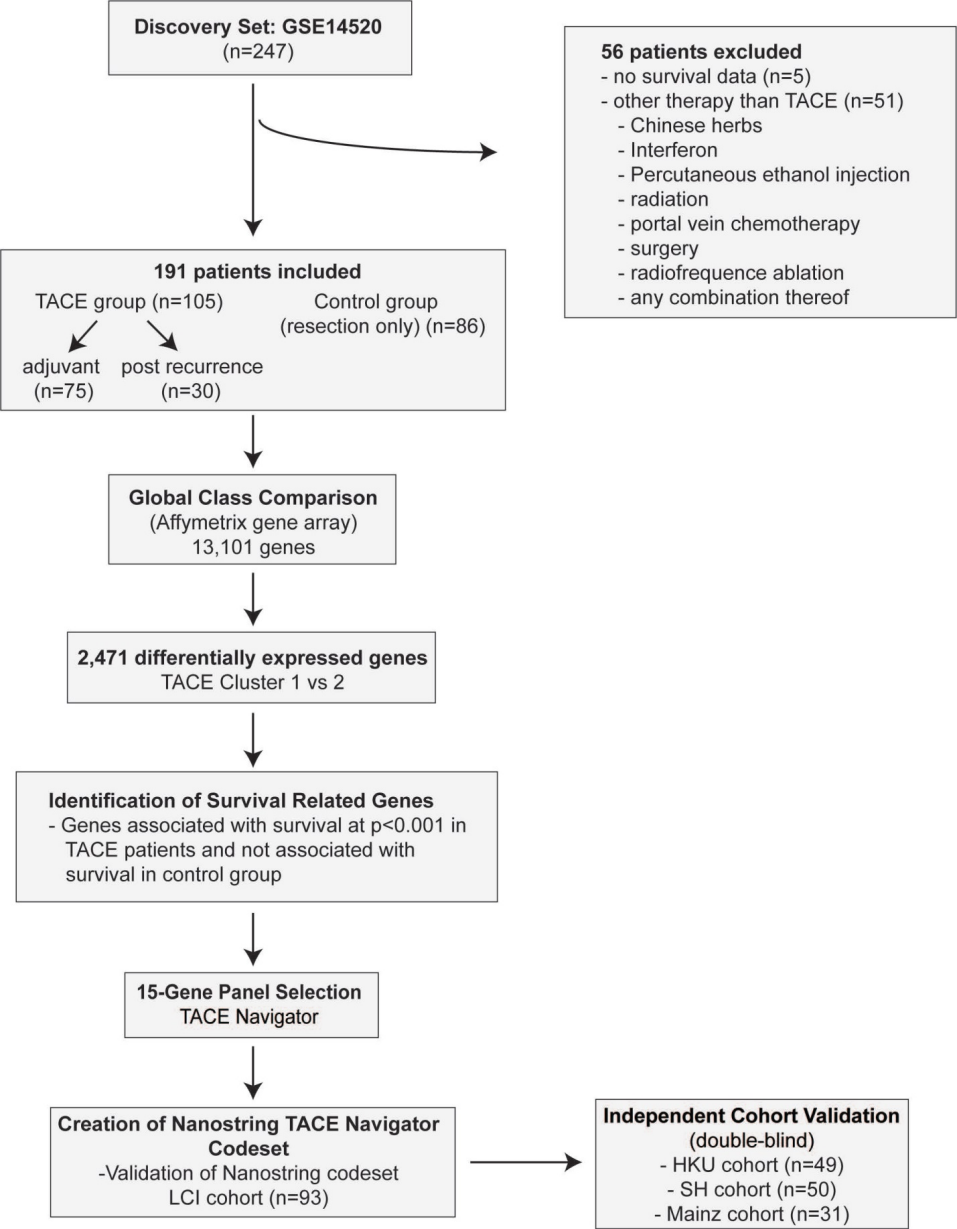
Study design for development of TACE Navigator including patient inclusion and exclusion criteria. Initial identification of gene signature identified using Affymatrix gene array including 13,101 genes in the LCI Cohort. Validation was performed utilizing NanoString platform in three additional TACE cohorts.

The LCI cohort includes 247 HCC patients who were prospectively recruited and underwent resection with curative intent at the Liver Cancer Institute and Zhongshan Hospital (Fudan University, China) between 2002 and 2003 20. Patients who were missing relevant clinical data (n=5), as well as those that underwent non-TACE adjuvant or recurrence therapy (n=56) were excluded for a total of 191 patients. The cohort which received TACE included a subset which received adjuvant TACE (adjuvant, n=75), as well as a subset which received TACE following tumor recurrence (post-recurrence, n=30). (Figure 1A, Table S1-3). All TACE patients from the LCI cohort received a combination of cisplatin, fluorouracil and mitomycin C.

### Validation Cohorts

We analyzed three independent cohorts to test our gene signature. The first cohort consisted of FFPE tumor samples from 49 patients with HCC who underwent curative intent resection with adjuvant TACE at the University of Hong Kong Medical Centre in Hong Kong. All patients in the Hong Kong cohort received cisplatin during the TACE procedure. The second cohort comprised FFPE tumor samples from 50 patients with HCC who underwent curative intent resection with adjuvant TACE at the Shandong Cancer Hospital and Institute in Jinan, Shandong Province, China. To ensure the stability of the test, sample preparation and analyses from this cohort were performed in a test laboratory at the Zhejiang University, China. For patients in the Shandong cohort, doxorubicin and cisplatin-based regimens were predominantly used. The third test cohort consisted of FFPE tumor samples from 31 patients with HCC who underwent TACE as primary therapy at Johannes Gutenberg University Medical Center in Mainz, Germany. For patients in the Mainz cohort, most patients received doxorubicin with drug-eluting beads (DEB TACE), while a minority of patients received TACE with Mitomycin C. All patients were treated and followed per the practices of their treating physicians. Clinical data was retrospectively collected for all patients.

To confirm that our gene signature is only applicable to patients undergoing TACE, a total of 112 HCC patients who did not receive TACE from two recently published cohorts (TIGER-LC Cohort, n=56; Korean Cohort, n=56) were also included as a negative control group 21,22.

### Development of Nanostring TACE Navigator Gene Signature

Bioinformatic analyses, including class comparison and survival risk prediction algorithms, were used to identify genes that were predictive of OS in the group receiving TACE, but not among those who received resection alone. All bioinformatic analyses were performed using BRB-ArrayTools (Bethesda, MD, USA). We developed a custom nCounter Gene Expression Codeset from NanoString (Seattle, WA, USA) consisting of a 15-gene TACE signature and six control genes. NanoString Digital Gene Expression Analysis was performed by the Center for Cancer Research Genomics Core in 93 of the TACE patients from the training/validation cohort, in which RNA was available. We developed a prognostic index equation prediction module based on the expression of each signature gene as measured by NanoString using the survival risk prediction function in BRB-ArrayTools, which we refer to as the TACE Navigator gene signature. Validation was performed using 10-fold cross validation.

Analyses for all test cohorts were performed in a blinded. NanoString analysis was performed and patients were assigned into predicted Responder or predicted Non-Responder groups using our prognostic index equation prediction module. Data were subsequently decoded and clinical data for each patient was obtained.

### Statistical analysis

Original processed gene expression data from the LCI cohort, as measured by Affymetrix, was obtained from GSE14520 at NCBI GEO (see supplementary methods). Gene expression as measured by NanoString counts, was Log2 transformed and then converted to Z-score within each cohort. In all statistical analyses, p<0.05 was considered statistically significant. Clinical data was evaluated using Fisher's exact test or 2-tailed Student's t-test. Patient survival was evaluated using Kaplan-Meier survival analysis with log-rank test. Correlation comparing Affymetrix gene expression and NanoString gene expression was evaluated using Pearson correlation. All statistics were calculated using GraphPad Prism 7.0 (San Diego, CA, USA).

Univariable and multivariable analyses were performed with Cox proportional hazards regression analysis using STATA 14.0 (College Station, TX, USA).

### Study oversight

This study was designed and conducted in strict accordance with REMARK guidelines 23. All tissue samples used in this study were obtained from patients who provided written informed consent. Institutional review boards at all study centers approved this study.

Additional descriptions of the methods are available in Supplemental Material.

## Results

### Association of Gene Expression with Clinical Outcome in TACE Patients

**Figure 2:**
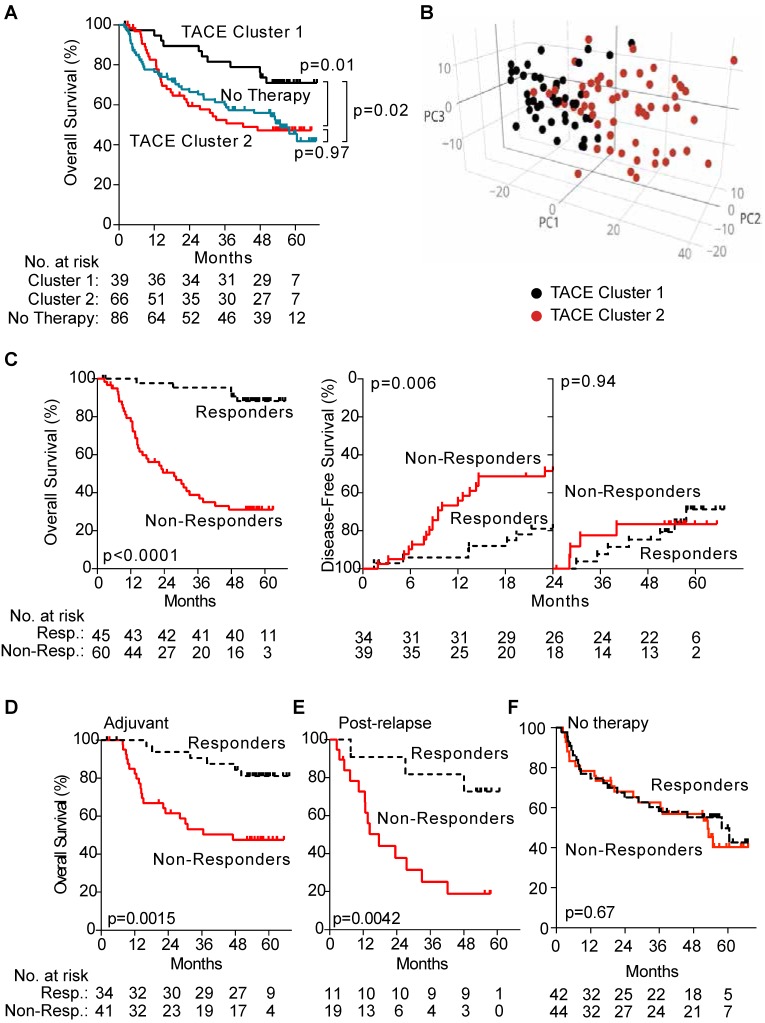
Panel A shows that when TACE patients from the test cohort are assigned to clusters by hierarchical clustering with the 1,292 most variable genes, there is a significant difference in OS between the two clusters, whereas no difference in OS is seen when comparing patients from TACE cluster 2 to patients receiving no additional therapy. Panel B depicts a principal component analysis in which TACE patients from the training/validation cohort are mapped based on the first three principal components of 13,101 global genes. Patients assigned to TACE cluster 1 (black dots) and TACE cluster 2 (red dots) show clear separation into unique groups. Panel C demonstrates that the 45 TACE patients assigned to the “Responder” cluster have significantly better OS and early (< 24 months) disease-free survival compared to the 60 patients assigned to the “Non-Responder” cluster. Panel D and E shows a significant difference in OS between patients assigned as Responders or Non-Responders is seen in the subset of 70 patients that received adjuvant TACE (panel D) and 30 patients that received post-recurrence TACE (Panel E). No difference in OS is seen in patients that received No Therapy when clustered by TACE Navigator, as seen in panel F. P values were calculated by log-rank test.

First, we identified the presence of subgroups among patients who received TACE by performing hierarchical clustering using a subset of unbiasedly selected “most variably expressed” genes from our global gene expression list, revealing two groups of patients (TACE cluster 1 and TACE cluster 2) that exhibited a significant difference in OS (Figure 2A). When TACE cluster 2 patients were compared to resection only patients, no statistically significant difference was noted (Figure 2A). These results indicate that a portion of TACE treated patients did not significantly benefit from treatment. In addition, multivariable analysis demonstrates that the survival benefit exhibited by TACE cluster 1 patients compared to patients receiving resection only disappears after correcting for clinical covariates (Table S4). These results indicate that enhanced stratification of patients is needed to determine if adjuvant TACE can offer a survival benefit.

Hierarchical clustering of TACE patients based on gene expression demonstrates that differences in tumor gene expression may be associated with TACE response. Consistently, principal component analysis with differentially expressed genes between TACE cluster 1 and TACE cluster 2 revealed the two clusters as distinct molecular groups with some overlap between patients was seen (Figure 2B). Thus, additional refinement of the two patient groups through the development of a gene signature to specifically predict TACE response, independent of other clinical variables, may serve to improve treatment outcomes.

### Development of a Gene Signature Associated with Overall Survival Following TACE Treatment

To develop a gene signature associated with survival following TACE, we followed a systematic approach (Figure 1). We first set out to identify genes in which there are notable differences in expression that can be quantitatively measured between TACE patients with better and worse OS. To do this we used class comparison to determine differentially expressed genes between TACE cluster 1 and cluster 2. We then performed survival risk prediction with Cox regression analysis to select only genes that are highly associated with OS. Finally, we eliminated genes that were associated with survival, as determined by survival risk prediction with Cox regression, in patients who did not receive TACE. This process yielded 15 genes that formed the basis of our TACE-response gene signature (Table S5).

Hierarchical clustering of TACE patients using this 15-gene signature revealed two groups of patients with significantly different OS (median OS >67 versus 39.5 months), which we designated as TACE Responders (n=45, 42.9%) and TACE Non-Responders (n=60, 57.1%), (Figure 2C). For the subset of 75 patients receiving only adjuvant TACE, a significant difference in early disease-free survival was seen between patients assigned as Responders and Non-Responders (Figure 2C). When the TACE patients were sub-divided into patients who received adjuvant TACE and re-clustered via hierarchical clustering, the 15-gene signature was capable of separating patients into two groups with a significant difference in OS (median OS >67 versus 47.1 months) (Figure 2D). Median OS in Responders receiving post-recurrence TACE was also higher compared to Non-Responders (>67 versus 17.6 months) (Figure 2E). As expected, these 15 genes did not separate patients into clusters with different OS in the resection only (p=0.67) cohort (Figure 2F).

Univariable analysis with Cox proportional-hazards regression demonstrated, in addition to the TACE Signature, that cirrhosis status, tumor size, microvascular invasion and tumor stage were each significantly associated with prognosis. Our final multivariable model showed that TACE Navigator, was independently associated with survival (Table 1). Additionally, TACE Responders demonstrated improved survival compared to resection only patients, even after correcting for clinical covariates (Table S6), indicating that stratification of patients receiving TACE is required in order to achieve a significant survival benefit.

### Validation and Testing of TACE Navigator Gene Signature

To develop the TACE Navigator gene signature as a prognostic device, we created a pipeline to validate and test our findings with a NanoString nCounter codeset as described in the methods. First, we ensured that gene expression as measured by Affymetrix correlated to gene expression as measured by NanoString and found that expression was highly correlated for all genes except GABARAPL3, which was subsequently removed from the signature (Figure S1). Removal of GABARAPL3 from the gene signature had no significant effect on patient assignment by hierarchical clustering, with only two patients who had initially been assigned as Responders re-assigned as Non-Responders (98% concordance).

**Figure 3:**
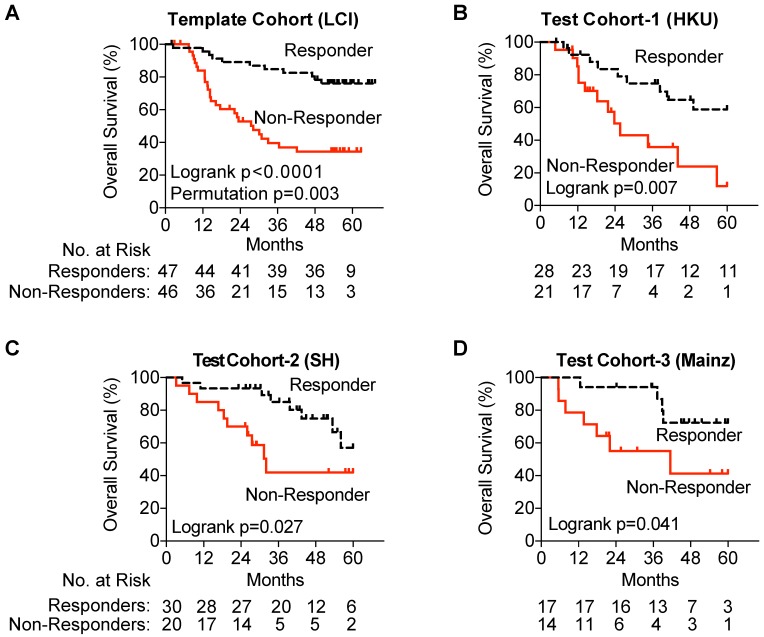
Validation of the TACE Navigator gene signature. Panel A demonstrates that in the test cohort, the 47 TACE patients assigned as “low risk” experience significantly better OS compared to the 46 TACE patients assigned as “high risk” by survival risk prediction using the TACE Navigator prognostic device. Panel B shows that when the device is used to predict TACE patient responders or non-responders in the Hong Kong test cohort, the 28 patients predicted as “Responders” experience significantly better OS than the 21 patients predicted to be “Non-Responders,” and Panel C shows when the device was examined in the Shandong test cohort, the 30 patients predicted as “Responders” experience significantly better OS than the 20 patients predicted to be “Non-Responders.” Panel D shows that for patients receiving palliative TACE in the Mainz test cohort, the 17 patients assigned to the “Responder” group experience significantly better OS than the 14 patients assigned to the “Non-Responder” group. P values were calculated by log-rank test. Permutation P value was calculated by survival risk prediction.

Gene expression was measured using NanoString, and patients were assigned into Responder and Non-Responder groups using the prognostic index equation and prognostic threshold created by our pipeline for the test cohort as well as each of the three validation cohorts. In our test cohort, utilizing NanoSting gene expression we once again demonstrated an association with improved survival amongst TACE Responders (p=0.0015) (Figure 3A). In each of the three test cohorts, a significant survival advantage was noted among TACE Responders vs Non-Responders (Hong Kong Cohort, median OS >60 vs 25.6 months; Shandong Cohort, median OS >61.3 vs 32.1 months; Mainz Cohort, median OS >60 vs 41.5 months) (Figure 3B-D).

Finally, as a negative control group, patients from the TIGER-LC and Korean cohorts who had not received TACE were assigned into predicted response groups using TACE Navigator (Figures S2A and S2B). No significant difference in OS was seen in patients assigned to either patient group (p=0.48 for TIGER-LC cohort; p=0.13 for Korean cohort).

### Molecular Signaling Associated with TACE Response

**Figure 4:**
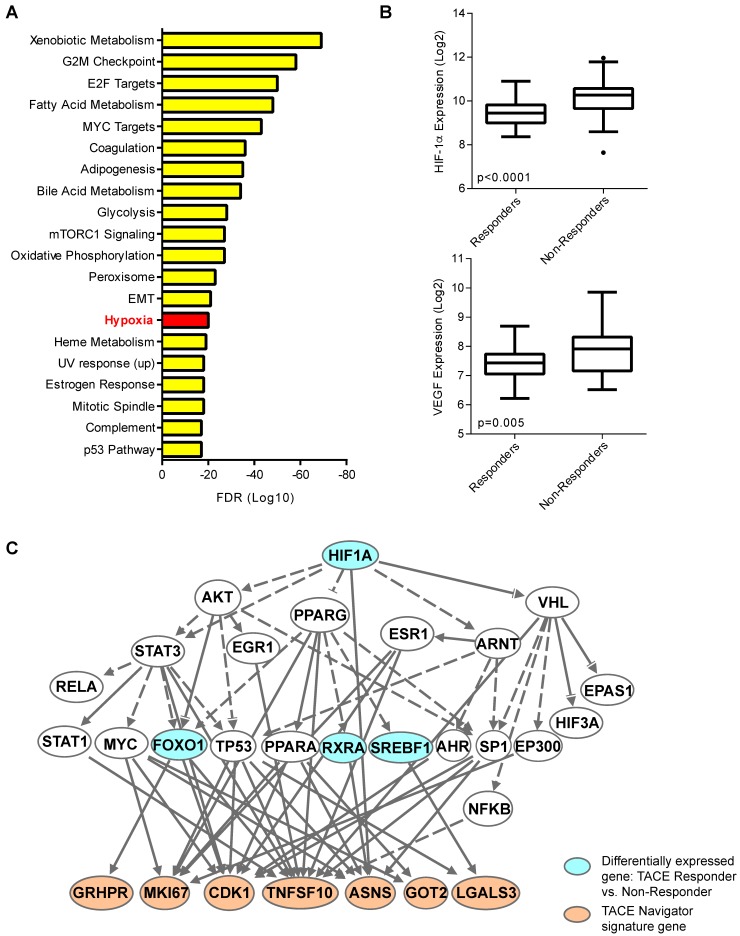
The hypoxia response may be linked to TACE treatment resistance. When all 1,726 differentially expressed genes between TACE Responders and Non-Responders are analyzed by Gene Set Enrichment Analysis by computing overlaps with the hallmark gene set molecular signatures, the hypoxia pathway is one of the top enriched pathways from this gene set, as shown in Panel A, and when examined directly, HIF-1α and target gene VEGF are up-regulated in TACE Non-Responders, compared to Responders, as shown in Panel B. When differentially expressed genes between TACE Responders vs. Non-Responders are input into Ingenuity Pathway Analysis, master hypoxia regulator HIF-1α is predicted to be directly upstream of seven TACE Navigator genes, as shown in Panel C. Bubbles shaded in blue indicate genes that are in the set of differentially expressed genes between TACE Responders and Non-Responders. Bubbles shaded in tan indicate genes from the TACE Navigator gene set. Box plots contain boxes extending from 25^th^ percentile to 75^th^ percentile, with the median value depicted by the line in the middle of the box, and Tukey whiskers (1.5 times Interquartile Range), with dots representing samples outside the Tukey variation. P values were calculated by Mann-Whitney U test.

Our results thus far indicated the presence of a molecularly-defined TACE-resistant subgroup of patients. To uncover potential mechanisms of TACE resistance, we examined pathways/networks by using global class comparison to determine which genes are differentially expressed between TACE Responders and Non-Responders from the training/validation cohort; inputting all 1,726 of these differentially expressed genes into Gene Set Enrichment Analysis; and computing overlaps with hallmark gene set molecular signatures. Genes known to be upregulated in response to hypoxia were enriched in this gene set (Figure 4A). We also compared the expression of hypoxia-inducible factor 1-alpha (HIF-1α) and classical hypoxia target gene vascular endothelial growth factor (VEGF) in Responders and Non-Responders. Indeed, both HIF-1α and VEGF are significantly up-regulated in Non-Responders (Figure 4B), indicating that the tumor microenvironment in Non-Responders may already be hypoxic prior to TACE, or may lead to an enhanced response to hypoxia induced during TACE. Ingenuity Pathway Analysis confirmed that HIF-1α may be connected directly to TACE response, as 7 of the 14 TACE Navigator signature genes are regulated directly downstream of HIF-1α signaling (Figure 4C). We also examined the expression of 155 HIF-1α targets that form the core response to hypoxia 24 (Table S7), and found a clear difference in gene expression in Responders and Non-Responders, indicating a connection between patient assignment by TACE Navigator and genes related to hypoxia (Figure S3A). Indeed, TACE patients with “low” hypoxic response (indicated by low HIF-1α and low VEGF expression) and TACE patients with “high” hypoxic response (indicated by high HIF-1α and high VEGF expression) form molecularly distinct clusters based on expression of the 155 hypoxia target genes, demonstrating a possible reprogramming of the hypoxic response in Non-Responder patients (Figure S3B). These results indicate that there is a likely difference in the activation of HIF-1α signaling between the two groups prior to receiving TACE, which may be a determinant in resistance.

## Discussion

In the era of precision medicine, in which predictive and prognostic biomarkers guide treatment based on molecular tumor features, advances for HCC treatment have lagged behind other cancer histologies. Guidelines drive clinical decision making for patients, and various treatments strategies including postoperative TACE, interferon, thymalfasin, and immunotherapy have been proposed. In this study, we aimed to design a clinically-relevant, TACE-specific prognostic device based on individualized patient gene expression in order to stratify patients who are most likely to benefit from TACE treatment. Although a number of HCC gene signatures have already been developed to predict outcome^25^, there are currently no gene signatures or biomarkers that have been specifically designed which are associated with survival in TACE patients. We developed and validated a TACE-specific 14-gene signature, TACE Navigator, which is independently associated with OS and early disease-free survival in a cohort of HCC patients from Asia. We then validated TACE Navigator, in a blinded manner, in three independent cohorts of patients: two cohorts from Asia in which patients had received adjuvant TACE, and one cohort from Europe in which patients had received TACE as primary therapy. This study provides a rationale for the continued investigation of TACE in both the adjuvant high risk setting as well as primary treatment in unresectable disease.

Examining differences in gene expression between TACE Responders and Non-Responders prior to treatment revealed that hypoxia response may be a potential mechanism of resistance. The induction of hypoxia through occlusion of the hepatic artery is a key component of TACE and leads to alteration of hypoxia induced genes. Other studies have demonstrated that when measured following the procedure, TACE induces changes in hypoxia master regulator HIF-1α and target gene VEGF 26, 27. However, to our knowledge, no study has examined potential differences in VEGF and HIF-1α gene expression in tumors in patients prior to receiving TACE and the associated treatment response. This study is the first to demonstrate that it is possible that in tumors of Non-Responder patients, the tumor microenvironment has undergone hypoxic reprogramming prior to treatment, as demonstrated by differences in expression of HIF-1α targets, yet the precise mechanism of TACE resistance remains unknown.

TACE is a highly heterogeneous procedure in which there is no standard of practice. Physicians have a number of options for TACE customization, including choice of chemotherapeutic agent, embolizing agent, and number of procedures or time between procedures 28. Patient selection bias may also influence the receipt of TACE. For our study, these factors remain a retrospective unknown. In addition, tumor samples are scarce given that no major guidelines recommend biopsy for this patient population. Therefore, we were forced to relay on limited banked tissue samples and retrospectively collected clinical data. Despite these potential confounders, TACE Navigator was capable of assigning cases into Responder and Non-Responder subtypes in cohorts from four independent centers with marked differences in both the procedure and patient population, indicating robustness of this device. The development of TACE Navigator as a tool to stratify patients to optimal treatment regimens represents the first milestone needed to conduct prospective clinical trials in gene expression analysis, and its application among different populations is a critical future direction. This technology represents a potential paradigm shift in TACE treatment selection in HCC.

## Supplementary Material

Supplementary figures and tables.

## Figures and Tables

**Table 1:** Univariable and multivariable cox model of clinical variables associated with overall survival in LCI cohort (n=105).

	Univariable		Multivariable	
Variable	Hazard Ratio (95% CI)	p value	Hazard Ratio (95% CI)	p value
TACE Navigator				
Responder	ref	-	ref	ref
Non-Responder	10.11 (3.95-25.86)	<0.001	9.56 (3.54 - 25.83)	<0.001
Age				
<50	ref	-	-	-
>50	1.04 (0.57- 1.89)	0.900	-	-
Sex				
Male	ref	-	-	-
Female	1.28 (0.40-4.14)	0.680	-	-
Hepatitis Status				
None	ref	-	-	-
Chronic Carrier	0.63 (0.19 - 2.08)	0.448	-	-
Active Viral Replication	0.96 (0.28 - 3.36)	0.954	-	-
Cirrhosis				
Negative	ref	-	ref	-
Positive	7.81 (1.07-56.82)	0.042	6.77 (0.90-51.07)	0.063
Child-Pugh Score				
A	ref	-	-	-
B	1.28 (0.48-3.42)	0.619	-	-
AFP				
≤400 ng/ml	ref		-	-
>400 ng/ml	1.34 (0.74-2.44)	0.333	-	-
Tumor Size				
<3 cm	ref	-	ref	-
≥3 cm	2.47 (1.14-5.34)	0.021	1.15 (0.49-2.71)	0.747
Multinodular				
No	ref	-	-	-
Yes	1.19 (0.59-2.42)	0.629	-	-
Microvascular Invasion				
No	ref	-	ref	-
Yes	1.76 (1.18-2.62)	0.006	2.38 (1.01-5.62)	0.046
TNM Stage				
I	ref	-	ref	-
II	2.18 (1.00-4.76)	0.049	0.63 (0.21-1.85)	0.400
III	4.26 (1.94-9.34)	<0.001	1.02 (0.37-2.78)	0.968

ref = reference variable

**Table 2:** Univariable and multivariable cox model of clinical variables associated with overall survival in Hong Kong (n=49).

	Univariable		Multivariable	
Variable	Hazard Ratio (95% CI)	p value	Hazard Ratio (95% CI)	p value
TACE Navigator				
Responder	ref	-	ref	ref
Non-Responder	3.16 (1.32-7.56)	0.01	2.54 (1.03 - 6.26)	0.043
Age				
<50	ref	-	-	-
>50	2.25 (0.86- 5.91)	0.100	-	-
Sex				
Male	ref	-	-	-
Female	0.94 (0.28-3.20)	0.922	-	-
Hepatitis Status				
None	ref	-	-	-
Chronic Carrier	3.73 (0.49 - 28.20)	0.448	-	-
Active Viral Replication	3.12 (0.36 - 26.88)	0.300	-	-
Cirrhosis				
Negative	ref	-	-	-
Positive	0.59 (0.26-1.36)	0.215	-	-
Child-Pugh Score				
A	ref	-	-	-
B	1.28 (0.48-3.42)	0.619	-	-
AFP				
≤400 ng/ml	ref		-	-
>400 ng/ml	1.78 (0.76-4.20)	0.185	-	-
TNM Stage				
I	ref	-	ref	-
II	1.51 (0.38-6.06)	0.562	1.49 (0.37-6.01)	0.572
III	4.49 (1.21-16.64)	0.025	3.65 (0.96-13.89)	0.058
ref = reference variable				
