# Supplementary material for: Gene signature predictive of hepatocellular carcinoma patient response to transarterial chemoembolization
Source: Int J Biol Sci. 2019 Oct 3;15(12):2654–63. doi: 10.7150/ijbs.39534 (PMC6854367; doi:10.7150/ijbs.39534)
Supplement: Supplementary file 1 — Supplementary figures and tables. [file ijbsv15p2654s1.pdf]

**Supplementary Material for Fako et al. Gene signature predictive of hepatocellular carcinoma patient response to transarterial chemoembolization**

## Supplementary Methods

## Clinical Specimens

*Test Cohort*

The training/validation cohort was derived from the Liver Cancer Institute (LCI) cohort in which a total of 247 HCC patients were prospectively recruited and underwent radical resection at the Liver Cancer Institute and Zhongshan Hospital (Fudan University) between 2002 and 2003. Microarray profiling of LCI cohort patients was performed previously. Briefly, gene expression using RNA extracted from flash-frozen tumor tissue were previously profiled using Affymetrix Human Genome U133 2.0 microarray platform in two formats, Affymetrix GeneChip HG-U133A 2.0 or 96 HT HG-U133A 2.0 microarray platform, each containing the same probesets, as described (NCBI GEO accession number GSE14520). Data were processed by combining the CEL files from the two Affymetrix series using the matchprobes package in the R programming environment. Thereafter, the RMA method in the R affy package was used to obtain probe set expression summaries. Both raw and processed data are available in the GSE14520 at NCBI GEO. This dataset contains 488 samples: 247 tumor samples and 239 non-tumor samples, with expression information of the 13,101 genes in which signal could be measured. Of the 488 tumor and non-tumor samples contained in this data set, all 247 patients with tumor tissue available were considered for this study. Archived RNA extracted from the flash-frozen tumor tissue was stored at -80°C.

All TACE patients from the LCI cohort received a combination of cisplatin, fluorouracil and mitomycin C. Of the remaining patients, 86 received no additional therapy (Resection Only) and 51 received other forms of therapy (Other Therapy), not including TACE, following surgical resection. Patients who were administered TACE as adjuvant therapy following resection were those who were deemed to have a high probability of relapse (e.g. tumor size > 10 cm; >1 tumor nodules; or with vascular invasion, etc.). In this context, TACE is used for both diagnosis and treatment, in which digital subtraction angiography is performed to identify any tumor staining in the liver following resection. If tumor stains are noted, the size, location and number of stains are evaluated and TACE treatment is performed with superselective catheterization. If no tumor stains are noted, 1/3 of the standard dose of chemotherapy and lipiodol are injected into the hepatic artery. Patients designated as Other Therapy did not receive TACE during their treatment. Following surgical resection, Other Therapy patients received portal vein chemotherapy, interferon alfa therapy, radiofrequency ablation, percutaneous ethanol injection, or traditional Chinese medicine, or a combination thereof, and were treated outside usual clinical guidelines.

*Validation*

In the Hong Kong test cohort, patients who received TACE were those who were judged to have a high risk of recurrence following resection by the operating surgeons. The presence of tumor vs. non-tumor tissue was verified by H&E staining, and tumor tissue was collected by scraping five 5µm tumor sections for each patient. Total RNA was isolated using the Roche High Pure FFPET RNA Isolation Kit (Indianapolis, IN) according to manufacturer’s instructions. All patients in the Hong Kong test cohort received cisplatin during the TACE procedure.

For the Shandong test cohort, patients who received TACE were those who were judged to have a high risk of recurrence following resection by the operating surgeons. The presence of tumor tissue was verified by H&E staining. Tumor tissue was collected by scraping five 10μm tumor sections for each patient. Total RNA was isolated using a MasterPure RNA Purification Kit (Epicenter, Madison, WI) according to manufacturer's instructions. For patients in the Shandong cohort, doxorubicin and cisplatin-based regimens were predominantly used.

For the Mainz test cohort, patients were treated with palliative TACE in accordance with BCLC guidelines. Total RNA was isolated using a peqGOLD Total RNA Kit (VWR, Darmstadt, Germany) according to manufacturer's instructions. For patients in the Mainz cohort, most patients received doxorubicin with drug-eluting beads (DEB TACE), while a minority of patients received TACE with Mitomycin C.

**Signature Development and Patient Assignment**

Bioinformatic analyses, including class comparison and survival risk prediction algorithms, were then used to identify genes that were predictive of overall survival in the group of 105 patients receiving TACE, but not in 86 other patients who received no additional therapy following resection. All bioinformatic analyses were performed using BRB-ArrayTools (Bethesda, MD).

TACE Navigator was developed using a custom nCounter Gene Expression Codeset from NanoString (Seattle, Washington), consisting of 15-signature genes and six control genes. NanoString Digital Gene Expression Analysis was performed by the Center for Cancer Research Genomics Core in 93 TACE patients from the training/validation cohort. A prognostic index equation prediction module based on the expression of each signature gene was created using the survival risk prediction function in BRB-ArrayTools. Validation was performed using 10-fold cross validation.

NanoString analysis was then performed in a double-blind manner in the test and verification cohorts. Gene expression, measured by NanoString counts, was Log2 transformed and then converted to Z-score within each cohort. Patients were assigned into predicted Responders or Non-Responder groups using the prognostic index equation. Data were subsequently decoded and clinical data for each patient was obtained.

**Univariable and Multivariable Analysis**

Univariable and multivariable analyses were performed with Cox proportional hazards regression analysis using STATA 14.0 (College Station, TX). The association of each clinical variable on survival was first evaluated with univariable analysis, followed by multivariable analysis, which included clinical variables that were significantly associated with survival in the univariable analysis. Age grouping was chosen by median age in the training/validation cohort. Alanine aminotransferase and alpha-fetoprotein groupings were chosen based on commonly used normal vs. abnormal clinical values. For TNM staging, stage I indicates a single tumor with no vascular invasion whereas stage II and greater indicates that multiple tumors or vascular invasion has taken place, thus groups II and III were grouped together. No multicollinearity of covariates was found, and the proportional hazards assumption was met in the final models.

**Supplementary Figure 1. Affymetrix expression of TACE Navigator genes is correlated to NanoString expression**

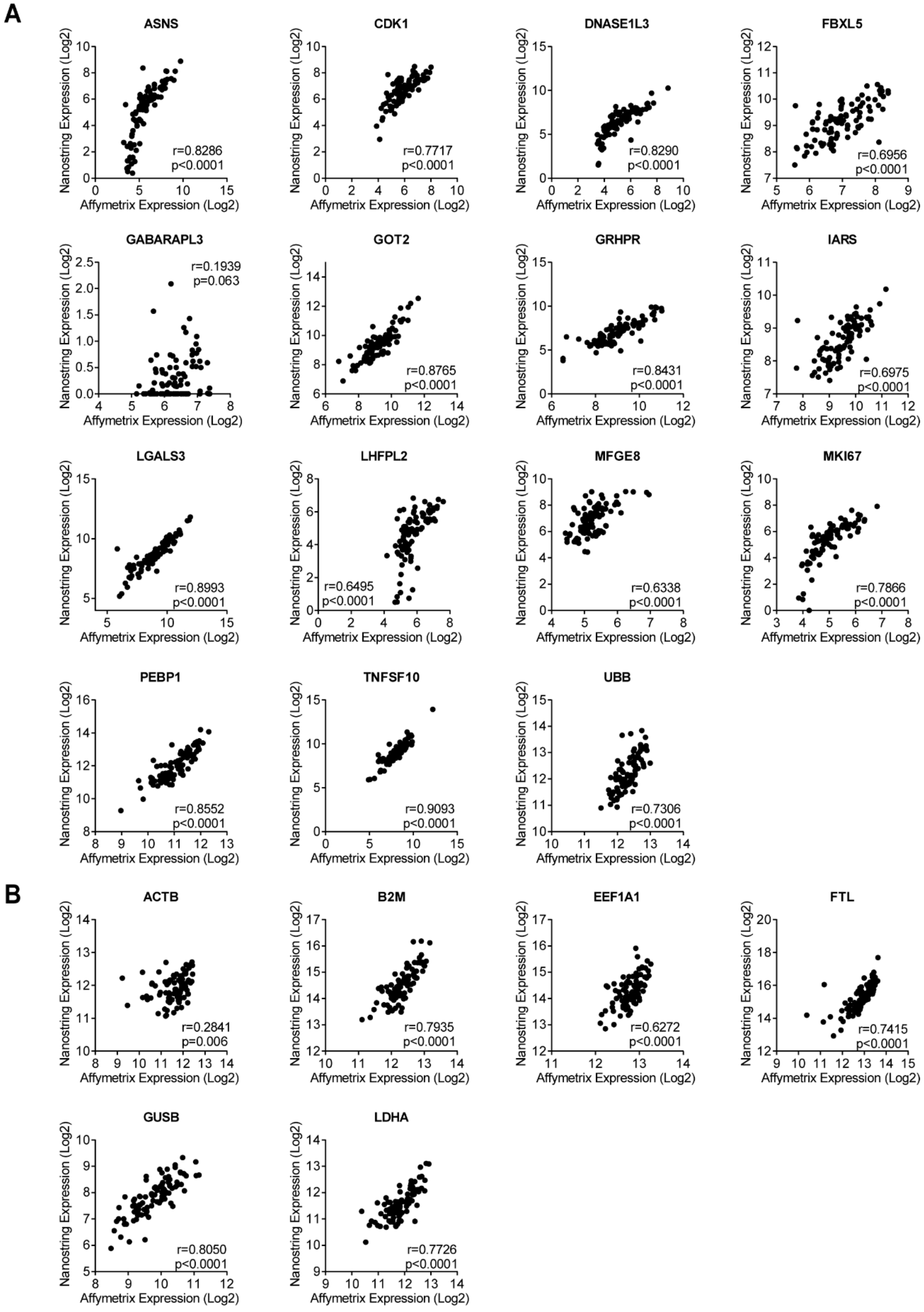

Correlation between gene expression (Log2), as measured by Affymetrix chip and NanoString, is shown for (A) TACE Navigator signature genes and (B) accompanying housekeeping genes. P and R values shown in each panel were calculated by Pearson Correlation, with a P value of less than 0.05 indicating statistical significance.

**Supplementary Figure 2. The TACE Navigator gene signature does not predict overall survival in patients who did not receive TACE**

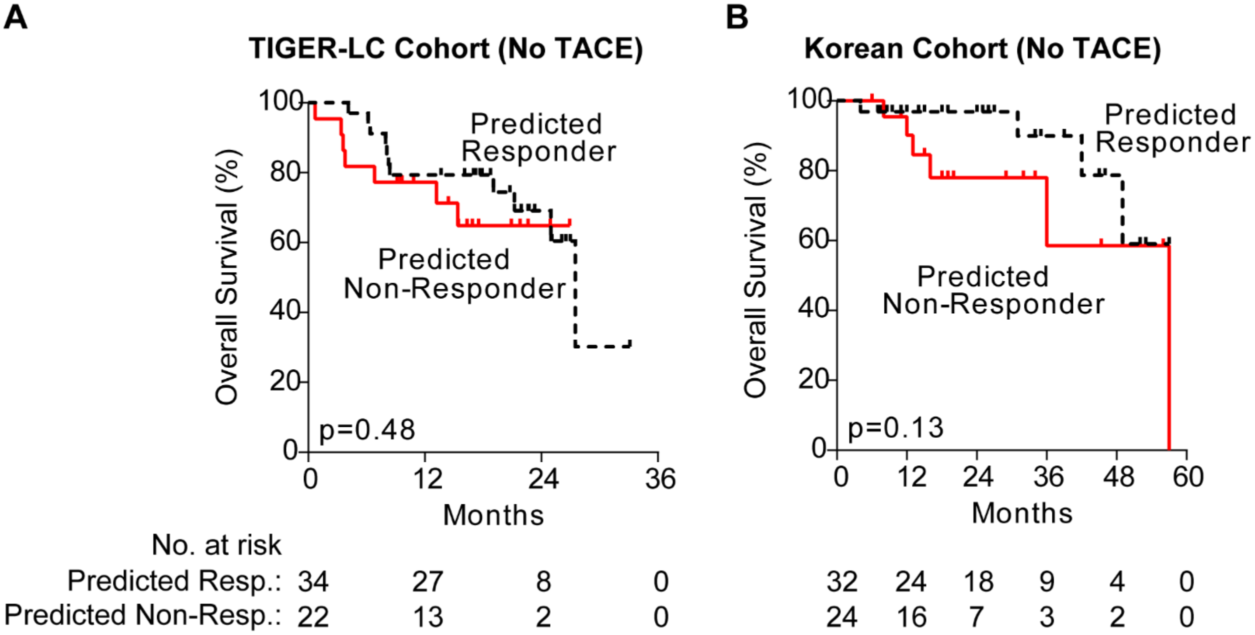


HCC patients from two independent cohorts who did not receive TACE: (A) TIGER-LC and (B) Korean Cohort were assigned into predicted Responder or Non-Responder groups using our developed prognostic index equation and prognostic threshold. In both cohorts, no significant difference in overall survival was seen in patients assigned to the two groups, as shown by Kaplan-Meier curve.

**Supplementary Figure 3. Responders and Non-Responders exhibit differential expression of hypoxia-related genes**

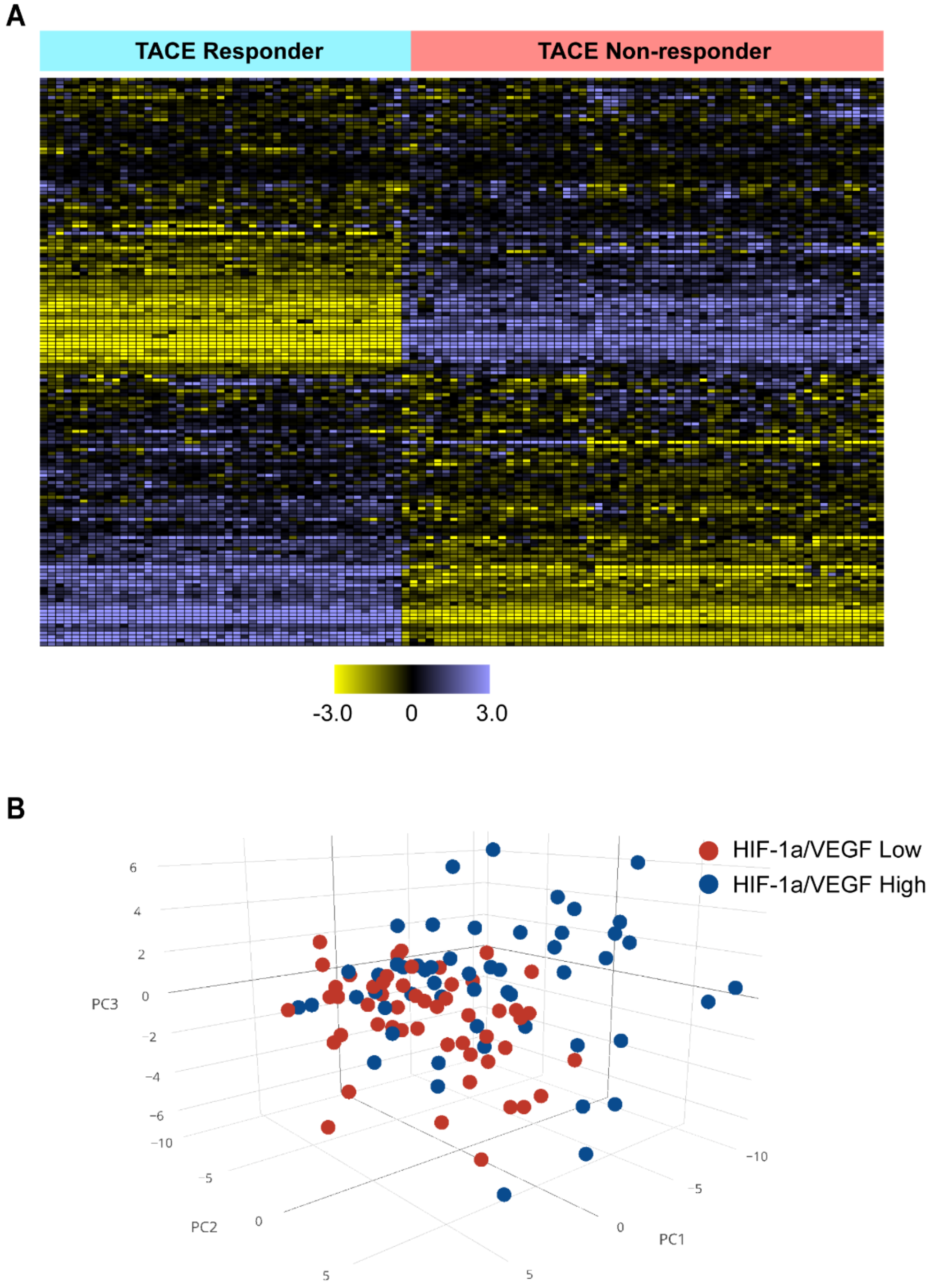


Heat map of 155 hypoxia target genes in TACE Responders and Non-Responders with columns representing individual patients and rows representing expression of each variable gene (A). Both patients and genes were clustered using Pearson Correlation distance and average linkage using the Genesis program. 100% concordance of TACE Responder and Non-Responder groups were observed following clustering. Expression values are Log2, and yellow indicates relative under-expression and purple indicates relative over-expression of each gene.

**Supplementary Table 1. Inclusion of patients from LCI cohort (GSE14520) and assignment into each therapy group**

| Adjuvant TACE subset (Included) | Post-Recurrence TACE subset (Included) | Resection Only (Included) | Other Therapy (Excluded) | Missing Survival Data (Excluded) |
|---|---|---|---|---|
| LCS_007A | LCS_008A | LCS_010A | LCS_002A | LCS_204A |
| LCS_009A | LCS_012A | LCS_014A | LCS_004A | LCS_283A |
| LCS_019A | LCS_023A | LCS_015A | LCS_005A | LCS_347A |
| LCS_020A | LCS_024A | LCS_016A | LCS_011A | X02_342A |
| LCS_025A | LCS_032A | LCS_018A | LCS_021A | X02_262A |
| LCS_027A | LCS_035A | LCS_022A | LCS_036A | |
| LCS_028A | LCS_067A | LCS_040A | LCS_039A | |
| LCS_029A | LCS_072A | LCS_041A | LCS_042A | |
| LCS_031A | LCS_088A | LCS_044A | LCS_054A | |
| LCS_033A | LCS_096A | LCS_045A | LCS_066A | |
| LCS_034A | LCS_120A | LCS_046A | LCS_074A | |
| LCS_038A | LCS_138A | LCS_048A | LCS_083A | |
| LCS_043A | LCS_139A | LCS_051A | LCS_089A | |
| LCS_047A | LCS_145A | LCS_056A | LCS_093A | |
| LCS_049A | LCS_178A | LCS_057A | LCS_095A | |
| LCS_050A | LCS_190A | LCS_061A | LCS_103A | |
| LCS_062A | LCS_194A | LCS_063A | LCS_107A | |
| LCS_065A | LCS_198A | LCS_064A | LCS_123A | |
| LCS_068A | LCS_200A | LCS_069A | LCS_125A | |
| LCS_071A | LCS_207A | LCS_073A | LCS_126A | |
| LCS_075A | LCS_224A | LCS_076A | LCS_129A | |
| LCS_079A | LCS_227A | LCS_078A | LCS_135A | |
| LCS_085A | LCS_234A | LCS_084A | LCS_143A | |
| LCS_086A | LCS_238A | LCS_090A | LCS_148A | |
| LCS_092A | LCS_267A | LCS_091A | LCS_149A | |
| LCS_097A | LCS_273A | LCS_094A | LCS_152A | |
| LCS_100A | LCS_274A | LCS_099A | LCS_153A | |
| LCS_104A | LCS_281A | LCS_101A | LCS_157A | |
| LCS_110A | LCS_333A | LCS_102A | LCS_162A | |
| LCS_116A | LCS_403A | LCS_105A | LCS_164A | |
| LCS_117A | | LCS_106A | LCS_173A | |
| LCS_118A | | LCS_108A | LCS_175A | |
| LCS_121A | | LCS_109A | LCS_182A | |
| LCS_127A | | LCS_119A | LCS_183A | |
| LCS_134A | | LCS_122A | LCS_188A | |
| LCS_136A | | LCS_130A | LCS_193A | |
| LCS_140A | | LCS_131A | LCS_195A | |
| LCS_142A | | LCS_132A | LCS_199A | |
| LCS_146A | | LCS_137A | LCS_201A | |
| LCS_154A | | LCS_144A | LCS_203A | |
| LCS_158A | | LCS_147A | LCS_206A | |
| LCS_159A | | LCS_150A | LCS_219A | |
| LCS_161A | | LCS_151A | LCS_230A | |
| LCS_166A | | LCS_156A | LCS_248A | |
| LCS_167A | | LCS_160A | LCS_250A | |
| LCS_170A | | LCS_163A | LCS_256A | |
| LCS_171A | | LCS_165A | LCS_277A | |
| LCS_177A | | LCS_169A | LCS_290A | |
| LCS_185A | | LCS_172A | LCS_339A | |
| LCS_191A | | LCS_174A | LCS_341A | |
| LCS_192A | | LCS_179A | LCS_401A | |
| LCS_196A | | LCS_180A | | |
| LCS_197A | | LCS_184A | | |
| LCS_208A | | LCS_189A | | |
| LCS_209A | | LCS_205A | | |
| LCS_212A | | LCS_210A | | |
| LCS_213A | | LCS_211A | | |
| LCS_223A | | LCS_215A | | |
| LCS_228A | | LCS_216A | | |
| LCS_231A | | LCS_222A | | |
| LCS_240A | | LCS_236A | | |
| LCS_241A | | LCS_237A | | |
| LCS_245A | | LCS_243A | | |

| | | | | |
|---|---|---|---|---|
| LCS_251A<br>LCS_259A<br>LCS_260A<br>LCS_263A<br>LCS_264A<br>LCS_265A<br>LCS_266A<br>LCS_270A<br>LCS_272A<br>LCS_284A<br>LCS_289A<br>LCS_393A | | LCS_247A<br>LCS_249A<br>LCS_253A<br>LCS_254A<br>LCS_261A<br>LCS_262A<br>LCS_268A<br>LCS_269A<br>LCS_275A<br>LCS_278A<br>LCS_279A<br>LCS_282A<br>LCS_285A<br>LCS_286A<br>LCS_291A<br>LCS_343A<br>LCS_344A<br>LCS_346A<br>LCS_400A<br>LCS_406A<br>LCS_415A<br>LCS_424A<br>LCS_426A | | |

**Supplementary Table 2. Clinical characteristics of LCI test cohort treatment groups**

| Variable | TACE (N=105) | Resection Only (N=86) | P Value* |
|---|---|---|---|
| Age—year | | | |
| Median | 50 | 50 | 0.71 |
| Range | 27-73 | 21-77 | |
| Sex—no. (%) | | | |
| Female | 8 (7.6) | 12 (14.0) | 0.16 |
| Male | 97 (92.4) | 74 (86.0) | |
| HBV—no. (%) | | | |
| Chronic carrier | 71 (67.6) | 63 (73.3) | 0.74 |
| Active virus | 28 (26.7) | 21 (24.4) | |
| Negative /Missing data | 6 (5.7) | 2 (2.3) | |
| Cirrhosis—no. (%) | | | 0.06 |
| No | 12 (11.4) | 3 (3.5) | |
| Yes | 93 (88.6) | 83 (96.5) | |
| Alanine aminotransferase—no. (%) | | | 0.14 |
| Normal (≤50 U/L) | 56 (53.3) | 55 (64.0) | |
| Elevated (>50 U/L) | 49 (46.7) | 31 (36.0) | |
| Alpha-fetoprotein—no. (%) | | | 0.99 |
| ≤200 ng/mL | 52 (49.5) | 43 (50.0) | |
| >200 ng/mL | 52 (49.5) | 42 (48.8) | |
| Missing Data | 1 (1.0) | 1 (1.2) | |
| Tumor Size—no. (%) | | | 0.99 |
| ≤3 cm | 30 (28.6) | 24 (27.9) | |
| >3 cm | 75 (71.4) | 62 (72.1) | |
| Microvascular Invasion—no. (%) | | | 0.23 |
| No | 68 (64.8) | 48 (55.8) | |
| Yes | 37 (35.2) | 38 (44.2) | |
| Multinodular Tumor—no. (%) | | | 0.60 |
| No | 84 (80.0) | 66 | |
| Yes | 21 (20.0) | 20 | |
| TNM Stage—no. (%) | | | 0.44 |
| I | 44 (41.9) | 29 (33.7) | |
| II+III | 56 (53.3) | 49 (57.0) | |
| Missing Data | 5 (4.8) | 8 (9.3) | |
| BCLC State—no. (%) | | | 0.86 |
| 0+A | 76 (72.4) | 58 (67.4) | |
| B+C | 24 (22.8) | 20 (23.3) | |
| Missing Data | 5 (4.8) | 8 (9.3) | |
| Survival (mo) | | | 0.22 |
| Median | >66.3 | 54.8 | |
| Range | 1.8->67 | 2.5->67 | |

*A P value of less than 0.05 was considered to indicate statistical significance. P values were calculated with the use of Fisher's exact tests, except for age, which was calculated with 2-tailed Student's t-test, and survival, which was calculated with the log-rank test.

**Supplementary Table 3. Clinical characteristics of test cohort TACE patients and test cohorts TACE patients**

| Variable | LCI Training/Validation Cohort (N=105) | Hong Kong Test Cohort (N=49) | Shandong Test Cohort (N=50) | Mainz Test Cohort (N=31) |
|---|---|---|---|---|
| Age—year | | | | |
| Median | 50 | 54 | 51 | 70 |
| Range | 27-73 | 24-74 | 31-71 | 57-91 |
| Sex—no. (%) | | | | |
| Female | 8 (7.6) | 7 (14.3) | 5 (10.0) | 26 (83.9) |
| Male | 97 (92.4) | 42 (85.7) | 45 (90.0) | 5 (16.1) |
| HBV—no. (%) | | | | |
| Chronic carrier | 71 (67.6) | 34 (69.4) | 42 (84.0) | 5 (16.1) |
| Active virus | 28 (26.7) | 9 (18.4) | 1 (2.0) | 0 (0.0) |
| Negative/Missing data | 6 (5.7) | 6 (12.2) | 7 (14.0) | 26 (83.9) |
| Cirrhosis—no. (%) | | | | |
| No | 12 (11.4) | 19 (38.8) | n.a. | 8 (25.8) |
| Yes | 93 (88.6) | 30 (61.2) | n.a. | 23 (74.2) |
| Alpha-fetoprotein—no. (%) | | | | |
| Negative (≤200 ng/mL) | 52 (49.5) | 24 (49.0) | 24 (48.0) | 14 (45.2) |
| Positive (>200 ng/mL) | 52 (49.5) | 24 (49.0) | 19 (38.0) | 9 (29.0) |
| Missing Data | 1 (1.0) | 1 (2.0) | 7 (14.0) | 7 (22.6) |
| Tumor Size—no. (%) | | | | |
| ≤3 cm | 30 (28.6) | 8 (16.3) | 19 (38.0) | 4 (12.9) |
| >3 cm | 75 (71.4) | 41 (83.7) | 29 (58.0) | 25 (80.6) |
| Missing Data | 0 (0.0) | 0 (0.0) | 2 (4.0) | 2 (0.05) |
| Microvascular Invasion—no. (%) | | | | |
| No | 68 (64.8) | 22 (44.9) | n.a. | 20 (64.5) |
| Yes | 37 (35.2) | 27 (55.1) | n.a. | 11 (35.5) |
| TNM Stage—no. (%) | | | | |
| I | 44 (41.9) | 11 (22.4) | 27 (54.0) | 16 (51.6) |
| II+III+IV | 56 (53.3) | 38 (77.6) | 23 (46.0) | 11 (35.5) |
| Missing Data | 5 (4.8) | 0 (0.0) | 0 (0.0) | 4 (12.9) |
| BCLC Stage—no (%) | | | | |
| 0+A | 24 (22.9) | n.a. | 32 (64.0) | 19 (61.3) |
| B+C | 75 (72.4) | n.a. | 18 (36.0) | 12 (38.7) |
| Missing Data | 5 (4.7) | n.a | 0 (0.0) | 0 (0.0) |
| Survival (mo) | | | | |
| Median | >67 | 44.1 | >60 | 59.1 |
| Range | 2.5->67.3 | 4.8->60 | 3.7->60 | 5.5->60 |

n.a. denotes data not available

**Supplementary Table 4. Hazard ratios for death among TACE Cluster 1 and Resection Only patients, according to univariable and multivariable analysis**

| Clinical Variable | Univariable Analysis<br><br>Hazard Ratio<br>(95 % CI) | P Value | Multivariable Analysis<br>Hazard Ratio<br>(95 % CI) | P Value |
|---|---|---|---|---|
| Treatment Group (TACE Cluster 1 vs. Resection Only)<br>TACE Cluster 1: 39 (31.2%)<br>Resection Only: 86 (68.8%) | 0.45 (0.23-0.88) | **0.019** | 0.66 (0.33-1.35) | 0.260 |
| Age (≤50 yr vs. >50 yr)<br>≤50 yr: 56 (44.8%)<br>>50 yr: 69 (55.2%) | 0.59 (0.35-1.02) | 0.058 | n.a. | |
| Sex (male vs. female)<br>Male: 111 (88.8%)<br>Female: 14 (11.2%) | 1.41 (0.56-3.56) | 0.453 | n.a. | |
| HBV (active virus vs. chronic carrier)<br>Active virus: 31 (24.8%)<br>Chronic carrier: 91 (72.8%)<br>Missing data/no virus: 3 (2.4%) | 1.82 (1.02-3.26) | **0.044** | 1.63 (0.85-3.13) | 0.143 |
| Cirrhosis (yes vs. no)<br>Yes: 118 (94.4%)<br>No: 7 (5.6%) | 1.65 (0.40-6.78) | 0.488 | n.a. | |
| Alanine aminotransferase (>50 U/L vs. ≤50 U/L)<br>Elevated (>50 U/L): 50 (40.0%)<br>Normal (≤50 U/L): 75 (60.0%) | 1.32 (0.77-2.28) | 0.309 | n.a. | |
| Alpha-fetoprotein (>200 ng/mL vs. ≤200 ng/mL)<br>>200 ng/mL: 53 (42.4%)<br>≤200 ng/mL: 71 (56.8%)<br>Missing data: 1 (0.8%) | 1.79 (1.04-3.10) | **0.036** | 1.09 (0.58-2.02) | 0.812 |
| Tumor size (>3 cm vs. ≤3 cm)<br>>3 cm: 84 (67.2%)<br>≤3 cm: 41 (32.8%) | 1.51 (0.84-2.72) | 0.170 | n.a. | |
| Microvascular invasion (yes vs. no)<br>Yes: 50 (40.0%)<br>No: 75 (60.0%) | 1.92 (1.12-3.29) | **0.018** | 2.38 (1.30-4.33) | **0.005** |
| Multinodular tumor (yes vs. no)<br>Yes: 25 (20.0%)<br>No: 100 (80.0%) | 1.79 (0.97-3.29) | 0.064 | n.a. | |
| TNM Stage (II+III vs. I)<br>II+III: 67 (61.6%)<br>I: 49 (39.2%)<br>Missing data: 9 (7.2%) | 2.69 (1.41-5.10) | **0.003** | n.a. | |
| BCLC Stage (B+C vs. 0+A)<br>B+C: 24 (19.2%)<br>0+A: 92 (73.6%)<br>Missing data: 9 (7.2%) | 3.44 (1.84-6.45) | **<0.001** | 2.77 (1.35-5.69) | **0.005** |

**Supplementary Table 5. 15 TACE Navigator genes**

| Gene Symbol | Description | Fold Change Responders vs. Non-Responders | Parametric p-value |
|---|---|---|---|
| ASNS | Asparagine synthetase | 0.33 | $<1x10^{-7}$ |
| CDK1* | Cyclin-dependent kinase 1 | 0.54 | $3.4x10^{-6}$ |
| DNASE1L3 | Deoxyribonuclease I-like 3 (DNase) | 2.91 | $<1x10^{-7}$ |
| FBXL5 | F-box and leucine-rick repeat protein 5 | 1.52 | $1.8x10^{-6}$ |
| GABARAPL3 | GABA(A) receptors associated protein like 3 | 1.41 | $8.5x10^{-6}$ |
| GOT2 | Glutamic-oxaloacetic transaminase 2, mitochondrial | 1.92 | $1x10^{-7}$ |
| GRHPR | Glyoxylate reductase/hydroxypyruvate reductase | 2.20 | $<1x10^{-7}$ |
| IARS | Isoleucyl-tRNA synthetase | 0.64 | $4x10^{-7}$ |
| LGALS3 | Lectin, galactoside-binding, soluble 3 | 0.35 | $<1x10^{-7}$ |
| LHFPL2 | Lipoma HMGIC fusion partner-like 2 protein | 0.57 | $<1x10^{-7}$ |
| MFGE8 | Milk fat globule-EGF factor 8 protein | 0.80 | $8.16x10^{-5}$ |
| MKI67 | Antigen Ki-67 | 0.63 | $<1x10^{-7}$ |
| PEBP1 | Phosphatidylethanolamine binding protein 1 | 1.74 | $<1x10^{-7}$ |
| TNFSF10 | Tumor necrosis factor superfamily, member 10 (TRAIL) | 2.05 | $1.7x10^{-6}$ |
| UBB | Ubiquitin B | 1.23 | $6.8x10^{-6}$ |

*Note: CDK1 is abbreviated as CDC2 in the training/validation cohort dataset.

**Supplementary Table 6. Hazard ratios for death among TACE Responders and Resection Only patients, according to univariable and multivariable analysis**

| Clinical Variable | Univariable Analysis Hazard Ratio (95 % CI) | P Value | Multivariable Analysis Hazard Ratio (95 % CI) | P Value |
|---|---|---|---|---|
| Treatment Group (TACE Responders vs. Resection Only) | 0.16 (0.06-0.41) | **<0.001** | 0.21 (0.08-0.55) | **0.001** |
| Resection Only: 86 (65.7%) | | | | |
| TACE Responders: 45 (34.3%) | | | | |
| Age (≤50 yr vs. >50 yr) | 0.64 (0.37-1.15) | 0.137 | n.a. | |
| ≤50 yr: 57 (43.5%) | | | | |
| >50 yr: 74 (56.5%) | | | | |
| Sex (male vs. female) | 1.24 (0.49-3.14) | 0.650 | n.a. | |
| Male: 116 (88.6%) | | | | |
| Female: 15 (11.4%) | | | | |
| HBV (active virus vs. chronic carrier) | 1.88 (1.00-3.54) | **0.049** | 1.59 (0.79-3.23) | 0.196 |
| Active virus: 29 (22.1%) | | | | |
| Chronic carrier: 99 (75.6%) | | | | |
| Missing data/no virus: 3 (2.3%) | | | | |
| Cirrhosis (yes vs. no) | 1.90 (0.46-7.83) | 0.375 | n.a. | |
| Yes: 122 (93.1%) | | | | |
| No: 9 (6.9%) | | | | |
| Alanine aminotransferase (>50 U/L vs. ≤50 U/L) | 1.45 (0.82-2.57) | 0.205 | n.a. | |
| Elevated (>50 U/L): 52 (39.7%) | | | | |
| Normal (≤50 U/L): 79 (60.3%) | | | | |
| Alpha-fetoprotein (>200 ng/mL vs. ≤200 ng/mL) | 2.03 (1.12-3.65) | **0.019** | 1.23 (0.64-2.37) | 0.532 |
| >200 ng/mL: 60 (45.8%) | | | | |
| ≤200 ng/mL: 69 (52.7%) | | | | |
| Missing data: 2 (1.5%) | | | | |
| Tumor size (>3 cm vs. ≤3 cm) | 1.45 (0.78-2.68) | 0.237 | n.a. | |
| >3 cm: 86 (65.7%) | | | | |
| ≤3 cm: 45 (34.3%) | | | | |
| Microvascular invasion (yes vs. no) | 0.75 (0.37-1.57) | 0.456 | n.a. | |
| Yes: 30 (22.9%) | | | | |
| No: 101 (77.1%) | | | | |
| Multinodular tumor (yes vs. no) | 2.14 (1.14-4.00) | **0.018** | 0.64 (0.26-1.61) | 0.348 |
| Yes: 26 (19.8%) | | | | |
| No: 105 (80.2%) | | | | |
| TNM Stage (II+III vs. I) | 2.68 (1.37-5.27) | **0.004** | n.a. | |
| II+III: 67 (51.1%) | | | | |
| I: 56 (42.7%) | | | | |
| Missing data: 8 (6.2%) | | | | |
| BCLC Stage (B+C vs. 0+A) | 4.65 (2.41-9.00) | **<0.001** | 3.99 (1.70-9.37) | **0.001** |
| B+C: 24 (18.3%) | | | | |
| 0+A: 98 (74.8%) | | | | |
| Missing data: 9 (6.9%) | | | | |

**Supplementary Table 7. Known and predicted HIF-1α targets used for TACE Responder and Non-Responder patient clustering**

| Known HIF-1α Targets: Expression Data Available | Known HIF-1α Targets: Expression Data Unavailable | Predicted Core Hypoxia Response Genes: Expression Data Available* | Predicted Core Hypoxia Response Genes: Expression Data Unavailable |
|---|---|---|---|
| ABCB1 | CYP2S1 | ABCF2 | ANKRD37 |
| ABCG2 | PROK1 | ACTR1A | GOPC |
| ADM | | ALDOC | LOC162073 |
| ADRA1B | | ANKRD37 | |
| AK3 | | ASCC1 | |
| ALDOA | | ASPH | |
| BHLHB2 | | ATF3 | |
| BHLHB3 | | ATF7IP | |
| BNIP3 | | BNIP3* | |
| BNIP3L | | C14orf169 | |
| CA9 | | C3orf28 | |
| CCNG2 | | CAD | |
| CD99 | | CCNB1 | |
| CDKN1A | | CLK3 | |
| CITED2 | | CRKL | |
| COL5A1 | | CXCR4* | |
| CP | | CYCS | |
| CTGF | | DDIT4* | |
| CTSD | | DHX40 | |
| CXCL12 | | EDEM3 | |
| CXCR4 | | EFNA1 | |
| DDIT4 | | EIF1 | |
| DEC1 | | EIF2B3 | |
| EDN1 | | ERO1L | |
| EGLN1 | | FH | |
| EGLN3 | | GADD45B | |
| ENG | | GAPDH* | |
| ENO1 | | GOPC | |
| EPO | | GOSR2 | |
| ETS1 | | GRK6 | |
| FECH | | GYS1 | |
| FN1 | | HIG2 | |
| FURIN | | ILF3 | |
| GAPDH | | INSIG2 | |
| GPI | | JMJD1A | |
| GPX3 | | JMJD2B | |
| HK1 | | JMJD2C | |
| HK2 | | KLF10 | |
| HMOX1 | | LDHA* | |
| HSP90B1 | | LOC162073 | |
| ID2 | | LOX* | |
| IGF2 | | MIF | |
| IGFBP1 | | MRPL4 | |
| IGFBP2 | | MRPS12 | |
| IGFBP3 | | MXI1* | |
| ITGB2 | | NARF | |
| KRT14 | | NDRG1 | |
| KRT18 | | NF2 | |
| KRT19 | | NR3C1 | |
| LDHA | | NXF1 | |
| LEP | | OXSR1 | |
| LOX | | P4HA1 | |
| LRP1 | | P4HA2 | |
| MCL1 | | PBEF1 | |
| MET | | PER2 | |
| MMP14 | | PGAM1 | |
| MMP2 | | PGK1* | |
| MXI1 | | PHLDA1 | |
| NOS2A | | PIM1 | |
| NOS3 | | PJA2 | |
| NPM1 | | PLOD1 | |
| NR4A1 | | PPME1 | |
| NT5E | | R3HDM1 | |

| | | | |
|---|---|---|---|
| PDGFA<br>PDK1<br>PFKFB3<br>PFKL<br>PGK1<br>PH-4<br>PKM2<br>PLAUR<br>PMAIP1<br>PPP5C<br>SERPINE1<br>SLC2A1<br>TERT<br>TF<br>TFF3<br>TFRC<br>TGFA<br>TGFB3<br>TGM2<br>TPI1<br>VEGFA<br>VIM | | RAB8B<br>RARA<br>RBPJ<br>RRAGD<br>RSBN1<br>SEC61G<br>SFRS7<br>SLC16A1<br>SLC7A6<br>SNRPD1<br>SPAG4<br>STC2<br>TGFBR1<br>TMEM45A<br>TPCN1<br>TUBG1<br>VDAC1<br>WSB1 | |

*Genes denoted with an asterisk indicate genes from the core hypoxia response that are previously known HIF-1α targets. Other genes in this column are predicted hypoxia response genes.
